# Left ventricular hypertrophy, geometric patterns and clinical correlates among treated hypertensive Nigerians

**DOI:** 10.4314/pamj.v4i1.53602

**Published:** 2010-03-04

**Authors:** Akintunde Adeseye, Akinwusi Olayinka, Opadijo George

**Affiliations:** 1 Division of Cardiology, Department of Internal Medicine, LAUTECH Teaching Hospital, Osogbo, Osun State, Nigeria.; 2 Cardiology Clinic, University Hospital of Tübingen, Eberhard Karls University, Tubingen, Germany

**Keywords:** hypertension, left ventricular geometry, clinical correlates, Nigeria

## Abstract

**Background:**

Left ventricular hypertrophy can be due to various reasons including hypertension. It constitutes an increased cardiovascular risk. Various left ventricular geometric patterns occur in hypertension and may affect the cardiovascular risk profile of hypertensive subjects.

**Methods:**

One hundred and eighty eight hypertensive participated in this study. Left ventricular hypertrophy was diagnosed by echocardiography. Relative wall thickness was derived by 2 × PWT/LVIDd. Subjects were arbitrarily categorized according to the duration of hypertension. Statistical analysis was done using SPSS 15.0.

**Results:**

The mean age of the study population was 55.95±10.71 years. Subjects who had hypertension for >5 years were more likely to be older and had a lower ejection fraction, larger left ventricular diastolic internal dimension than those with duration of hypertension <5 years. Concentric remodeling was the commonest left ventricular geometric pattern among the hypertensive subjects closely followed by normal left ventricular geometry. Concentric hypertrophy and eccentric hypertrophy were rare among the study population.Left ventricular geometry was associated mainly with left ventricular chamber and wall dimensions.

**Conclusion:**

Concentric remodeling is the commonest pattern of left ventricular geometric pattern of the left ventricle among hypertensive subjects. Left ventricular geometry is associated with the chamber and wall dimensions. Eccentric hypertrophy is associated with the lowest left ventricular systolic function and therefore possibly an herald to progressive systolic impairment.

## Background

Left venticular hypertrophy (LVH) and abnormal LV geometry are both important markers of cardiovascular risk among hypertensive subjects. They are associated with increased cardiovascular morbidity due to progressive ischaemic compromise, systolic and /or diastolic dysfunction, arryhthmias and sudden cardiac death [1–5]. LVH is defined as increase in left ventricular mass. It is usually associated with increase in wall tension, wall thickness or left ventriclar cavity size. There is usually no increase in the cavity size until later when there may be accompanying volume overload [6, 7].

LVH can be diagnosed by electrocardiography or echocardiography [8]. Though the sensitivity of various ECG criteria remains very low (ranging from 7 to 35% in mild hypertension and 10 to 50% in moderate and severe hypertension), [9] it is still in use in many parts of the world. However,Echocardiography, though not widely available in many parts of the developing countries, remains the more sensitive and acceptable modality for diagnosing LVH [10]. According to the Framingham’s study, a 40% rise in the risk of major cardiovascular events can be expected for each 39 g/m^2^ or standard deviation increase of left ventricular mass [11]. Left ventricular hypertrophy in hypertension is associated with increased prothrombotic state, microalbuminuria, higher systolic hypertension, increased body mass index, fasting serum lipids and blood sugar levels [12–15]. Left ventricular mass and left ventricular mass index more than two standard deviations of normal is defined as Echo LVH. One of the echocardiographic criteria for LVH are 134 and 110 g/m^2^ in men and women respectively, although there is a relatively wide range of published cutoff values [16, 17]. Findings from the Framingham Heart Study also suggested that normalization to height might be more accurate; the partition normal values are 163 g/m for men and 121 g/m for women [18]. Other studies have suggested different thresholds of 145 g/m in men and 120 g/m in women [19].

Various left ventricular geometrical pattern occurs as a result of adaptation of the left ventricle to increasing wall tension, pressure and volume changes in hypertension. The geometric patterns have significant impact on systolic and diastolic function of the left ventricle [1]. The geometric pattern of the left ventricle is therefore also important in cardiovascular prognosis. Four types of LV geometry have been described based on relative wall thickness (RWT) and left ventricular mass (LVM). They are: Normal geometry (normal RWT and LVM), concentric remodelling (Normal LVM and increased RWT), concentric hypertrophy (increased RWT and LVM), and eccentric hypertrophy (normal RWT and increased LVM). Patients with concentric remodelling may equally have increased adverse cardiovascular risk as those with concentric hypertrophy [20]. The aim of this study was to study the pattern of left ventricular hypertrophy and geometry among treated hypertensive and associated clinical correlates.

## Method

One hundred and eighty-eight consecutive hypertensive subjects on treatment for hypertension who had echocardiography seen at the LAUTECH Teaching Hospital, Osogbo, Nigeria were recruited for this study. Blood pressure was measured with Accosson sphygmomanometer and hypertension was diagnosed when systolic and/or diastolic blood pressure remained persistently over 140/90 mmHg respectively on more than two occasions after at least five minutes rest and/or the use of antihypertensive therapy.

All of them were receiving antihypertensive therapy consisting of at least two of a combination of diuretics, calcium channel blocker, Angiotensin converting enzyme inhibitors or beta blockers. Subjects with diabetes mellitus and chronic kidney disease were also excluded. Appropriate history taking, urinalysis and fasting blood sugar were used to exclude diabetes and chronic renal disease which are the commonest cause of secondary hypertension in our environment. The weight was taken using a standard weighing scale to the nearest 0.5kg while the height was taken with a stadiometer. The body mass index was derived by dividing the weight (in kilograms) by the square of the height (in meters). General physical examination was conducted on each participant. The duration of the hypertension was taken as the duration from the time a subject was told that he/she had elevated blood pressure to the time of the procedure. Subjects were categorized as “early” hypertensives if the duration of illness is less than one year. Those with duration more than 5 years were categorized as “long term” hypertensive subjects and those in between categorized as “intermediate”.

Echocardiography was done according to the recommendations of the American Society of Echocardiography (ASE) with the patient in the left lateral decubitus position [21]. Two dimensional and M-mode echocardiography were preformed according to standard criteria and an average of three values was taken. The M- mode derived parasternal view was used to assess for the chamber and wall dimensions of the left ventricle, including the derived systolic indices such as ejection fraction and fractional shortening. The left ventricular mass was derived using the Devereux modified ASE cube formula [22] which has been shown to correlate with necropsy findings. LVM= LV mass (g) = 0.8 (1.04 (IVSd + LVIDd + PWTd) 3 – (LVIDd)3 )+ 0.6.

Left ventricular hypertrophy was considered present if the left ventricular mass index is ≥ 134g/m^2^ and 110g/m^2^ for males and females respectively. Relative wall thickness was derived by 2 × PWT/LVIDd (LVIDd- left ventricular internal diastolic dimension, PWT-posterior wall thickness). Normal relative wall thickness was defined as < 0.45. Normal geometry was defined as normal left ventricular mass and normal relative wall thickness. Concentric remodeling was defined as increased relative wall thickness with normal left ventricular mass while concentric hypertrophy was defined as increased relative wall thickness and increased leftventricular mass. Eccentric hypertrophy was defined as increased left ventricular mass with normal relative wall thickness. Ethical approval was obtained for the study.

Statistical analysis was done using the Statistical Package for Social Sciences (SPSS) 15.0 (Chicago Ill.) Categorical variables were expressed as proportions and percentages while continuous variables were expressed as means ± standard deviation. Comparison between categorical variables were done by the use of chi square test, while that of numerical variables were done with the use of analysis of variance (MANOVA) test. A probability of p< 0.05 was taken as statistically significant.

## Results

One hundred and eighty-eight hypertensive Nigerians were recruited consecutively for this study. They were aged between 32 and 86 years old with mean age 55.95 ± 10.71 years. The study population consisted of 113 males (60.11%)and 75 females (39.89%). Long-term hypertensive subjects were older, and also had a statistically significantly lower ejection fraction and a larger left ventricular internal dimension in systole than those with duration of hypertension < 5 years. Compared with early hypertensive subjects, intermediate and long term hypertensive subjects had a higher relative wall thickness and posterior wall thickness. Left ventricular mass and left ventricular mass index was higher among those whose hypertension is > 1 year. (Table 1)

**Table 1:** Clinical and Echocardiogarphic parameters among the study population

Parameter	Early	Intermediate	Long term	p
Age	52.17± 11.01	55.4±10.8	59.33±9.36	<0.001**
BSA	1.8±0.17	1.79±0.19	1.85±0.18	0.127
SBP	145.2±26.2	145.8±20.8	148.4±23.6	0.700
DBP	88.36±11.32	89.8±12.5	89.24±11.3	0.799
EF	72.42±9.0	67.3±1.5	65.3±1.71	0.024**
FS	36.01±1.05	33.8±1.07	33.0±1.0	0.23
RWT	0.52±0.15	0.70±0.9	0.6±0.45	0.206
LVM	97.02±14.84	100.3±35.12	102.6±22.89	0.424
LVMI	53.69±8.7	53.8±8.3	55.9±14.1	0.515
LVIDs	29.42±8.90	29.82±9.24	33.65±10.57	0.023**
LVIDd	45.83±8.87	44.3±9.09	47.55±8.57	0.138
PWTd	11.48±2.08	14.74±1.84	12.95±1.06	0.36

** Statistically significant. BSA-Body surface area, SBP-Systolic blood pressure, DBP-Diastolic blood pressure, EF-Ejection fraction, FS-Fractional shortening, RWT-Relative wall thickness, LVM-Left ventricular mass, LVMI-Left ventricular mass index, LVIDs-Left ventricular end systolic dimension, LVIDd-Left ventricular end diastolic dimension, PWTd-Posterior wall thickness in diastole.

About 36% of those with “early” hypertension had normal geometry compared to about 23.6% of those with hypertension >1 year. A large part of those subjects with intermediate and long term duration of hypertension had concentric remodeling. (63.3% of intermediate duration and 64.1% of long term hypertensives) as compared to 52.46% of those who are recently diagnosed with hypertension. Abnormal left ventricular geometries are commoner among intermediate and long term hypertensive subjects than those recently diagnosed with hypertension. (Table 2)

**Table 2:** Left ventricular Geometric Patterns among the Study Population

Geometric pattern	Early hypertensives	Intermediate hypertensives	Long term hypertensives
Normal	22 (36.1%)	11(22.4%)	19(24.4%)
Concentric remodelling	32(52.4%)	31(63.3%)	50(64.1%)
Eccentric hypertrophy	3(4.9%)	2(4.1%)	5(6.4%)
Concentric hypertrophy	4(6.6%)	5(10.2%)	4(5.1%)
**Total**	**61**	**49**	**78**

Concentric remodelling was the commonest pattern of left ventricular geometry among these cohort and seem to be more commoner among male hypertensive subjects.LVM, LVMI, and RWT was highest among hypertensive subjects with concentric hypertrophy followed by those with eccentric hypertrophy as shown in Table 3. The table also shows that hypertensive subjects with eccentric hypertrophy had the highest left ventricular internal dimension in diastole compared to others while posterior wall thickness and interventricular septal thickness was higher among those with concentric remodelling and hypertrophy. Blood pressure was higher among those with concentric hypertrophy and concentric remodelling than those with normal geometry and eccentric hypertrophy. (Table 3)

**Table 3:** Demographic and echocardiographic parameters accross the different geometric patterns of the Left Ventricle.

Variable	Normal	Concentric remodelling	Concentric hypertrophy	Eccentric hypertrophy	P
AGE(years)	52.95±10.1	56.76±10.9	55.00±11.1	60.33±11.4	0.110
MALE(n)	25(13.3%)	79(42.0%)	6(3.2%)	3(1.6%)	0.038**
FEMALE(n)	27(14.3%)	33(17.6%)	8(4.3%)	7(3.7%)	
LVM(g)	99.15±11.22	93.89±12.33	169.6±78.95	128.29±14.09	<0.001**
LVMI(g/m2)	53.44±6.53	52.16±6.75	86.49±29.73	76.08±11.98	<0.001**
RWT	0.38±0.06	0.62±0.17	0.83±1.33	0.37±0.073	<0.001**
LVIDd(mm)	49.33±5.91	42.53±6.79	55.63±9.49	64.44±8.90	<0.001**
LVIDs(mm)	31.49±8.90	28.79±7.03	40.13±12.07	52.17±14.33	<0.001**
IVSD(mm)	11.75±2.35	14.8±8.63	12.43±4.76	11.66±2.72	<0.001**
PWTD(mm)	9.35±1.60	12.73±2.12	14.43±5.12	11.52±1.42	<0.001**
BSA(/m2)	1.86±0.18	1.81±0.19	1.71±0.11	1.78±0.13	0.366
SBP(mmHg)	137.39±20.3	151.95±23.72	154.3±23.7	136.4±26.09	0.001**
DBP(mmHg	89.10±11.49	90.76±11.86	90.0±18.25	83.0±8.23	0.09
EF (%)	67.92±17.56	70.3±12.52	58.23±24.96	52.0±15.6	0.001**
FS (%)	34.88±12.30	35.06±8.97	31.12±9.30	22.71±9.54	0.003**

** -Statistically significant. LVM-Left ventricular mass, LVMI-left ventricular mass index, RWT-Relative wall thickness, LVIDs-Left ventricular end diastolic dimension, LVIDs-Left ventricular end systolic dimension, IVSD-interventricular septal thickness, PWTd- Posterior wall thickness in diastole, BSA-Body surface area, SBP-Systolic blood pressure, DBP-Diastolic blood pressure, EF-Ejection fraction, FS-Fractional shortening.

## Discussion

Left ventricular geometry and structural alterations occurs in response to systemic hypertension in various pattern. This is determined to a large extent by whether pressure or volume overload is predominating [22–23]. Concentric remodeling and concentric hypertrophy may predominate in “early” and “intermediate” hypertensives due to the predominating pressure overload whereas eccentric hypertrophy progressively takes over with increased left ventricular mass due to increase in volume overload [23].

This study shows that the majority of the hypertensive subjects in this study had concentric remodeling. About three-fifths (60.1%)of the hypertensive subjects had concentric remodeling as against 27.7% with normal left ventricular geometry. A study of the pattern of left ventricular geometry among newly diagnosed essential hypertensives also documented that 28% of newly diagnosed subjects had normal geometry [24]. The similarity in the proportion of those with normal geometry between these two studies may suggest that antihypertensive therapy induce a reversal or at least stop the progression of left ventricular adaptive changes associated with increasing wall and volume tension to some extent. This may be consequent upon reduction of blood pressure or specific effect on left ventricular remodelling by specific antihypertensive therapy. It is therefore likely that antihypertensive therapy also have an influence on the geometric pattern in hypertensive subjects.

Effective and tight blood pressure control may retard or even reverse the adverse changes in cardiovascular geometry and dysfunction. Angiotensin converting enzyme inhibitors (ACE-I) and Angiotensin receptor blockers (ARBs) are particularly useful in this case as they have been shown to produce neurohormonal regulation, cardiovascular and renal remodeling than other groups of antihypertensive drugs. Although, other antihypertensive drugs also have influence on cardiac remodeling, control of blood pressure, and reversal of cardiac remodeling associated with pressure and volume overload in hypertensive subjects, ACE-I and ARBs are particularly useful [25]. Many of these subjects are not recently diagnosed hypertensive and were possibly expected to have a more ‘advanced’ geometry than concentric remodeling.

Progressive alteration in geometric pattern of the left ventricle also influence left ventricular systolic function parameters [26]. As the severity of abnormal geometry progresses, most of the derived systolic indices reduces, although they are still within normal limits. Those with eccentric hypertrophy had the lowest echocardiography derived systolic function. This may suggest that the beginning of progressive deterioration of global systolic function occurs in eccentric hypertrophy. Some reports have suggested that eccentric hypertrophy heralds a progressive prevalence of congestive cardiac failure among hypertensive subjects [27]. This study did not show a significant association of age with left ventricular geometric pattern although abnormal left ventricular geometry tend to occur among elderly individuals where cardiovascular risk factors are more likely to cluster and age associated blood pressure and vascular resistance are more likely to predominate. Other authors have shown a significant association with age among the Caucasian population [28].

Systolic blood pressure was associated with abnormal LV geometry and LVH in this study. Hypertensive subjects with concentric hypertrophy and concentric remodeling had the highest mean systolic blood pressure while those with eccentric hypertrophy had a similar mean blood pressure as those with normal left ventricular geometry [29]. This was despite similar blood pressure pattern among the “early”, “intermediate” and “long term” hypertensive subjects. This further suggest that chamber geometry may play an important role in determination of left ventricular systolic function rather than the systolic blood pressure alone [26]. Males in this study had a higher proportion of abnormal left ventricular geometry (concentric remodeling) despite treatment. The contribution of gender to left ventricular geometry may be as a result of genetic, gender specific response to hypertension or impact of other cardiovascular risk factors such as obesity.

Among these cohort, eccentric hypertrophy and concentric hypertrophy were equally common in agreement with other studies [30]. Left ventricular mass increased with increasing systolic blood pressure among the study participants especially among the males in agreement with similar studies [31, 32]. Systolic blood pressure determines the maximal wall tension and therefore, not surprising that it is associated with left ventricular mass.

## Conclusion

This study showed that concentric remodeling is the commonest type of geometric pattern present among treated hypertensive subjects. The presentation is associated with clinical parameters such as age, gender and duration of hypertension. Longitudinal studies are necessary to delineate the possible role of these findings in the adaptive mechanism of Left ventricular structure and on the cardiovascular morbidity pattern in hypertensive subjects.

## Competing interests

The authors declared no conflicts of interests whatsoever.

## Authors’ contributions

**AA**: study concept, design, data collection, statistical analysis, manuscript writing, and review. **AO**: study design, data collection, manuscript writing and review. **OG**: study design, manuscript writing, editing, review
